# Nicotinamide enhances myelin production after demyelination through reduction of astrogliosis and microgliosis

**DOI:** 10.3389/fncel.2023.1201317

**Published:** 2023-08-17

**Authors:** Stefanos Ioannis Kaplanis, Despoina Kaffe, Niki Ktena, Andriani Lygeraki, Ourania Kolliniati, Maria Savvaki, Domna Karagogeos

**Affiliations:** ^1^Department of Basic Science, School of Medicine, University of Crete, Heraklion, Greece; ^2^Institute of Molecular Biology and Biotechnology, Foundation for Research and Technology Hellas, Heraklion, Greece; ^3^Department of Biology, University of Crete, Heraklion, Greece; ^4^Laboratory of Clinical Chemistry, Medical School, University of Crete, Heraklion, Greece; ^5^Department of Pediatrics, Medical School, University of Crete, Heraklion, Greece

**Keywords:** caloric restriction, myelin, remyelination, microglia, astrocytes, nicotinamide (NAM)

## Abstract

Caloric restriction is the chronic reduction of total caloric intake without malnutrition and has attracted a lot of attention as, among multiple other effects, it attenuates demyelination and stimulates remyelination. In this study we have evaluated the effect of nicotinamide (NAM), a well-known caloric restriction mimetic, on myelin production upon demyelinating conditions. NAM is the derivative of nicotinic acid (vitamin B3) and a precursor of nicotinamide adenine dinucleotide (NAD^+^), a ubiquitous metabolic cofactor. Here, we use cortical slices *ex vivo* subjected to demyelination or cultured upon normal conditions, a lysolecithin (LPC)-induced focal demyelination mouse model as well as primary glial cultures. Our data show that NAM enhances both myelination and remyelination *ex vivo*, while it also induces myelin production after LPC-induced focal demyelination *ex vivo* and *in vivo*. The increased myelin production is accompanied by reduction in both astrogliosis and microgliosis *in vivo*. There is no direct effect of NAM on the oligodendrocyte lineage, as no differences are observed in oligodendrocyte precursor cell proliferation or differentiation or in the number of mature oligodendrocytes. On the other hand, NAM affects both microglia and astrocytes as it decreases the population of M1-activated microglia, while reducing the pro-inflammatory phenotype of astrocytes as assayed by the reduction of TNF-α. Overall, we show that the increased myelin production that follows NAM treatment *in vivo* is accompanied by a decrease in both astrocyte and microglia accumulation at the lesion site. Our data indicate that NAM influences astrocytes and microglia directly, in favor of the remyelination process by promoting a less inflammatory environment.

## 1. Introduction

Myelin is a lipid-rich multilayer membrane which surrounds the majority of vertebrate axons, provides them with metabolic and trophic support and insulates them, thus ensuring the rapid propagation of nerve signals. In the central nervous system (CNS) myelin is produced by oligodendrocytes (OLs) (Nave and Werner, 2014). Malfunction of the CNS myelinating glia, in particular mature oligodendrocytes, or their precursors, the oligodendrocyte precursor cells (OPCs), results in myelin disruption, which can lead to axonal demyelination and eventually contribute to axonal degeneration (Chen et al., 2022). Following demyelination in the CNS, new myelin sheaths are generated through the homeostatic process of remyelination by newly differentiated OLs (Franklin and Ffrench-Constant, 2008; Neely et al., 2022). However, remyelination is usually insufficient to recapitulate the original myelin ultrastructure (Orthmann-Murphy et al., 2020).

Apart from the obvious role of OLs in myelination and remyelination after an insult, other glial cell types such as microglia and astrocytes are important in these processes (Nave and Werner, 2014; Kuhn et al., 2019; Lloyd and Miron, 2019; Molina-Gonzalez and Miron, 2019; Djannatian et al., 2023). Regarding microglia, several studies have provided evidence that they are implicated in developmental myelination (Domingues et al., 2016). Moreover, microglia contribute to myelin plasticity by pruning myelin sheaths depending on neuronal activity (Hughes and Appel, 2020; McNamara et al., 2023). In response to CNS injury, microgliosis takes place, during which microglia become activated, change from a ramified to an amoeboid morphology and proliferate at the lesion site (Li and Zhang, 2016; Baaklini et al., 2019; Kalafatakis and Karagogeos, 2021). Activated microglia expand, migrate and accumulate within the damaged area playing both beneficial and detrimental roles regarding myelin repair (Amor et al., 2022). Briefly, they can be classified as M1 pro-inflammatory or M2 anti-inflammatory cells. The former promote inflammation and oligodendrocyte damage by secreting pro-inflammatory cytokines, while the latter promote myelin repair by producing trophic factors and removing debris through phagocytosis (Kotter et al., 2006; Hanisch and Kettenmann, 2007; Plemel et al., 2013; Skripuletz et al., 2013; Luo et al., 2017; Quan et al., 2022). In order for remyelination to be initiated and be successful, a switch from M1 to M2 phenotype is necessary (Miron et al., 2013; Lombardi et al., 2019; He et al., 2020).

In addition to microglia, astrocytes are also necessary for myelin formation and maintenance (Liedtke et al., 1996). However, their role on remyelination can also be either beneficial or detrimental. Upon CNS demyelination, astrocytes become activated and undergo a series of morphological and functional changes in a process known as reactive astrogliosis to form the glial scar which inhibits remyelination (Bannerman et al., 2007; Fan et al., 2018; Molina-Gonzalez and Miron, 2019; Fan and Huo, 2021; Quan et al., 2022). Astrocytes produce extracellular matrix molecules, such as fibronectin and N-cadherin, which contribute to remyelination failure as well (Stoffels et al., 2013). Furthermore, they can contribute to demyelination by promoting a pro-inflammatory response, secreting factors such as TNF-α and IL-6 (Cammer and Zhang, 1999; Itoh et al., 2018). On the other hand, astrocytes are necessary for remyelination to occur as they express factors known to be involved in OPC proliferation and differentiation (Franklin et al., 1991; Houben et al., 2020). Importantly, astrocyte activation results in microglia recruitment and efficient myelin debris clearance. Inactivation of astrocytes results in reduced microglia recruitment, reduced myelin debris clearance and thus reduced remyelination efficiency (Skripuletz et al., 2013).

In parallel, several recent studies have revealed that caloric restriction (CR) attenuates demyelination and stimulates remyelination (Mojaverrostami et al., 2020). CR refers to the chronic reduction of total caloric intake without malnutrition. CR in experimental autoimmune encephalomyelitis (EAE) mice, a well-established model of demyelination, decreases CNS inflammation and demyelination and enhances axonal integrity (Piccio et al., 2008). In addition, there is evidence that CR can promote remyelination in the corpus callosum (CC) of cuprizone-induced demyelinated mice *in vivo* (Mojaverrostami et al., 2020). Caloric restriction mimetics (CRMs) constitute autophagy inducers that mimic the biochemical and functional effects of CR, acting through the stimulation of deacetylation of cellular proteins (Madeo et al., 2014; Yang and Zhang, 2020). Recently, several studies implicated autophagy in myelin maintenance and axonal integrity (Park et al., 2018; Bankston et al., 2019; Aber et al., 2022; Ktena et al., 2022).

In this study we have evaluated the effect of nicotinamide (NAM), a well-known CRM, on myelin production upon demyelinating conditions. NAM is the derivative of nicotinic acid (vitamin B3) and a precursor of nicotinamide adenine dinucleotide (NAD^+^), which is a ubiquitous metabolic cofactor participating in numerous metabolic pathways (Schultz and Sinclair, 2016; Hou et al., 2021; Langley et al., 2021; Wang et al., 2021). Although in recent years effects of NAD^+^ and its precursors on remyelination have been observed, the main focus has been placed on oligodendrocytes (Ma et al., 2022), while much less is known about its effect on other glial types. A recent study identified NAD^+^ in astrocytes as a key player to reduce inflammation and favor remyelination *in vivo* after depletion of CD38, the main NAD^+^-depleting enzyme in CNS (Langley et al., 2021). Also, NAD^+^ supplementation results in reduced neuroinflammation in Alzheimer’s disease (Hou et al., 2021) and in EAE mice (Wang et al., 2021). As both astrocytes and microglia are key players in the CNS, we focus on the effect of NAM administration on these two populations.

## 2. Materials and methods

### 2.1. Animals

All animals used in this study were of C57BL/6 background (MGI Cat# 5657942, RRID:MGI_5657942, UK) and their number was selected following the 3R principle. They were kept at the animal facility of the Institute of Molecular Biology and Biotechnology, under temperature-controlled conditions in a 12 h light/dark cycle, fed by standard chow diet and water *ad libitum*. The animal facility of the Institute of Molecular Biology and Biotechnology (IMBB)- Foundation for Research and Technology Hellas (FORTH) (license nos. EL91-BIObr-01 and EL91-BIOexp-02) complies with all regulations and standards outlined in the Presidential Decree 56/30.04.2013 (Greek Law). All procedures were performed in accordance with the EU directives and regulations (2010/63/EU and L 276/33/20.10.2010) that are equivalent to NIH standards established by the Animal Welfare Acts and the documents entitled “Principles for Use of Animals” and “Guide for the Care and Use of Laboratory Animals” from the Office of Laboratory Animal Welfare. Experimental animal protocols have been approved with license number 93164 (AΔA: 73XB7ΛK-ΦIΣ).

### 2.2. *Ex vivo* experiments

#### 2.2.1. Cortical slices

P4 (postnatal day 4) C57BL/6 mouse pups were decapitated and their brains were dissected in cold aCSF medium [NaCl 125 mM, KCl 3.5 mM, NaHCO3 26 mM, C_20_H_36_O_2_ 1.26 mM, D-(+)-Glucose monohydrate 10 mM and MgCl_2_ hexahydrate 3 mM] without Ca^2+^, on ice. Subsequently, the brains were mounted on a vibratome (Leicabiosystems, Germany) and 300 μm coronal cortical slices were collected in ice cold aCSF medium. The slices were immediately transferred onto Millicell cell culture inserts (30 mm, hydrophilic PTFE, 0.4 μm, Merck-Millipore, PICM0RG50, Germany) using a transferring pipette, in a six-well plate containing warm organotypic medium [50% DMEM (Glutamax™, 4.5 g/L d-Glucose, -Pyruvate, Gibco, Cat# 61965-026, USA), 25% Horse Serum (Gibco, Cat# 26050088), 25% EBSS (Gibco, Cat# 24010043), 2% Penicillin/Streptomycin (Gibco, Cat# 15070063) and 0.5% Amphotericin B (Gibco, Cat# 15290018)]. All the medium was removed from the upper side of the membrane before the plate was placed into the incubator. The slices were kept in culture for 14 days at 37°C and 5% CO_2_. The medium was changed every other day. Demyelination of the slices was obtained by adding LPC at a concentration of 0.5 mg/ml for 15 h on day 14. After this period, the medium was replaced with preheated organotypic medium without LPC. The treatment began after LPC removal and lasted for 6 days. LPC used for these studies was not fluorescently labeled (Sigma-Aldrich, Cat# L1381, USA). For the NAM-treated group, nicotinamide (Sigma-Aldrich, Cat# N0636, USA) was diluted in 100% EtOH (Scharlau, Cat# ET0016005P, Spain) in order to prepare a stock solution of 0.1M NAM. The final concentration of NAM was 0.1 mM in 0.01% EtOH in organotypic medium. The medium was changed by replacing half of it every other day. For the control group we used organotypic medium with 0.01% EtOH. After 6 days of treatment the slices were fixed and prepared for immunofluorescent labeling. Multiple tissue slices can be placed on each Millicell insert in a six well plate. As cortical slices are rather large, we placed 3- 4 on a single insert.

#### 2.2.2. Slice culture immunofluorescence

Cortical slices were washed once with 1x PBS before they were fixed with 4% paraformaldehyde (PFA) (Sigma-Aldrich, Cat# 16005, USA) in PBS for 1 h at RT. The slices were subsequently rinsed in 1x PBS and blocked in 3% Horse Serum (Gibco, Cat# 26050088), 2% BSA, (PanReac AppliChem, Cat# A1391, Germany), and 0.5% Triton X-100 in 1x PBS for 2 h at RT. Following blocking, the slices were incubated for 48 h with primary antibodies at 4°C. The slices were then washed 3 times for 30 min with blocking solution and incubated with 4′,6-diamidino-2-phenylindole (DAPI) (1:1,500, Invitrogen, Cat# MP01306, USA) and the appropriate secondary antibodies overnight at 4°C. The slices were washed again 3 times with blocking solution for 30 min and finally mounted on a glass microscope slide using mounting medium containing Mowiol^®^ 4-88 Reagent (Millipore, Cat# 475904, Germany). The primary antibodies used were as follows: against myelin basic protein (rat monoclonal anti-MBP, MCA409S, BioRad, 1:300, USA), Neurofilament-H (chicken anti-NF200, Abcam, Cat# ab4680 1:10,000, UK). Secondary antibodies were all purchased from Life Technologies (Life Technologies, 1:1,000, USA). Images were obtained using confocal laser scanning microscopy (Leica microsystems, TCS SP8, Germany) and processed using Fiji image processing package.^1^

### 2.3. *In vivo* experiments

#### 2.3.1. LPC-induced demyelination mouse model *in vivo*

In this study we used the LPC demyelinating mouse model in order to study the effect of nicotinamide (NAM) on remyelination *in vivo*. The protocol used to induce focal demyelination with LPC injection was based on that published by Ferent et al. (2013) and was standardized with few modifications. Male C57BL/6 mice of 9–10 weeks of age were weighted prior to the experimental procedure and received anesthesia of ketamine/xylazine (per g of body weight: 100 μg of ketamine and 15 μg of xylazine) by intraperitoneal injection. After checking responsiveness to painful stimuli by pinching of the tail and hind limb, each animal was fixed on the stereotactic frame, by means of a mouse nose clamp adaptor and ear bars, so that their head position assured an identical height of the bregma and the lambda as well as of the right and left hemisphere. Following exposure of the skull and creation of a small opening with the use of a drill, 1 μL of either the vehicle solution (1x sterile PBS) or the LPC (Sigma-Aldrich, Cat# L1381, USA) solution (1% w/v LPC in 1x sterile PBS) was injected in the corpus callosum at a flow of 0.1 μL/min [anteroposterior axis (AP): +1 mm from the bregma, mediolateral axis (ML): −1 mm from the bregma, dorsoventral axis (DV): −2.2 mm from the dura]. Afterward, animals were carefully removed from the frame, sutured and left to recover in a clean cage with excess of food and water. We used one control (vehicle-treated) group and two groups treated with different NAM (Sigma-Aldrich, Cat# N0636, USA) concentrations (40 or 400 mg/kg/day of NAM in Serum). NAM was administrated in LPC-injected animals intraperitoneally once daily for 7 or 14 days starting from the day following the stereotactic surgery. Mice were sacrificed the day after the treatment completion. The dose concentration was selected according to previous studies regarding NAM (Uddin et al., 2017; Xie et al., 2020).

#### 2.3.2. Tissue fixation

For euthanasia, mice received an intraperitoneal injection of anesthesia (per g of body weight: 200 μg of ketamine and 30 μg of xylazine) and were transcardially perfused with 20 ml of 1x PBS followed by cold 25 ml 4% PFA (Sigma-Aldrich, Cat# 16005, USA) in 1x PBS. Forebrains were carefully dissected and post-fixed in 4% PFA in 1x PBS at 4°C, for 30 min.

#### 2.3.3. Tissue preparation for immunohistochemistry

Following post-fixation, tissues were washed three times in 1x PBS and were immersed in 30% sucrose, 0.1% NaN3 in 1x PBS. They were kept at 4°C until the tissues sank at the bottom of the container. After cryoprotection, samples were embedded in a gel containing 15% w/v sucrose and 7.5% gelatin from porcine skin (Sigma-Aldrich, Cat# G2500, USA) in 1x PBS. To ensure uniform freezing, samples were submerged in methylbutane and frozen at −35 to −40°C. Tissue blocks were then stored at −80°C before proceeding to cryosectioning (Leicabiosystems, Germany). A total of 15-μm-thick coronal sections were collected on superfrost plus glass slides (Thermo scientific) and stored at −20°C until further processing.

#### 2.3.4. Immunohistochemistry protocol

Cryosections were encircled in Dako Pen (Cat# S200230, Dako, Agilent Technologies, USA) and post-fixed in ice-cold acetone at −20°C for 10 min. After three 5-min washes in 1x PBS, they were incubated in Blocking Solution [5% bovine serum albumin (fraction V, BSA), (PanReac AppliChem, Cat# A1391, USA), 0.5% Triton X-100 in 1x PBS] for 1 h at RT. Subsequently, they were incubated with the appropriate primary antibody diluted in Blocking Solution at 4°C, overnight. The cryosections were then washed 3 times in 1x PBS and were incubated with DAPI and the appropriate secondary fluorescently labeled antibodies in Blocking Solution for 2 h at RT. After washing 3 times with 1x PBS they were mounted using mounting medium containing Mowiol^®^ 4-88 Reagent (Millipore, Cat# 475904, Germany). Slides were kept at 4°C until imaging took place or at −20°C for long term storage. The primary antibodies used were as follows: anti-MBP (1:200, rat, Serotec Cat# MCA409S, RRID:AB_325004), anti-PDGFRa (1:100, rat, Millipore, Cat# CBL1366, Germany RRID:AB_11211998), anti-CC1 (1:100, mouse, Calbiochem Cat# OP80-100UG), anti-IBA-1 (1:500, rabbit, Invitrogen, Cat# PA5-21274, USA), anti-GFAP (1:2,000, mouse, Sigma-Aldrich, Cat# G3893, USA, RRID:AB_477010). Secondary antibodies were all purchased from Molecular probes (Life Technologies 1:800, USA). For nuclear staining, DAPI (1:1,500, Invitrogen, Cat# MP01306, USA) was used. Images were obtained using confocal laser scanning microscopy (Leica microsystems, TCS SP8, Germany) and processed using Fiji image processing package (see text footnote 1).

### 2.4. *In vitro* experiments

#### 2.4.1. Microglia, oligodendrocytes and astrocytes primary cultures

Primary mixed glial cell cultures were prepared from the cortices of male and female P2 (postnatal day 2) C57BL/6 mouse pups, as previously described (Eigel et al., 2019). In short, cortices were dissected and, after the removal of meninges, they were trypsinized and triturated with the cell suspension being, finally, transferred in poly-D-lysine (PDL, Sigma-Aldrich, Cat# P7405, USA, 0.01 mg/ml)-coated 75 cm^2^ culture flasks. Cells were cultured in DMEM (GlutaMAX™, 4.5 g/L d-Glucose, -Pyruvate, Gibco, Cat# 61965-026), supplemented with 10% FBS (Gibco, Cat# 10437028) and 1% Penicillin/Streptomycin 10,000 units (Gibco, Cat# 15140-122) in an incubator with 5% CO_2_ at 37°C. The culture medium was replenished twice a week. After 12–14 days, when a clear, confluent layer of cells was formed, the mixed glial culture was separated into different cell populations according to their ability to attach to the flask.

At first, microglial cells were isolated using an orbital shaker at 200 rpm for 1 h at 37°C. Medium was removed from the flasks, microglia were centrifuged at 300 *g* for 10 min and after resuspension, they were seeded at an initial density of 150,000 cells per well in 24-well plates or at an initial density of 75,000 cells per well in 48-well plates containing 9 mm glass coverslips. All plates were previously coated overnight with poly-D-lysine (PDL, as above). Microglial cells were maintained in DMEM, supplemented with 10% FBS and 1% Penicillin/Streptomycin. The day of cell plating is considered as DIV0. At DIV1 cells cultured on glass coverslips were treated with 100 ng/ml of lipopolysaccharides (LPS) (InvivoGen, Cat# tlrl-3pelps) and medium was removed 24 h later. Afterward, NAM (Sigma-Aldrich, Cat# N0636, USA, diluted in 70% EtOH) was added to the wells at three different concentrations (0.1, 0.2, and 0.4 mM) and cells were fixed at DIV5 for immunocytochemistry assays. On the other hand, cells cultured in 24-well plates were treated with NAM at DIV0, at three different concentrations (0.1, 0.2, and 0.4 mM). At DIV3 they underwent starvation and were maintained in starvation medium (DMEM supplemented with 0.1% FBS and 1% Penicillin/Streptomycin) for 4 h. Afterward, starvation medium was removed and cells were treated with 100 ng/ml of LPS and different NAM concentrations (0.1 mM, 0.2 mM and 0.4 mM) in DMEM, supplemented with 10% FBS and 1% Penicillin/Streptomycin for 24 h, until they were collected for lysis. In another case, microglial cells cultured in 24-well plates were treated with bafilomycin A1 (Sigma-Aldrich, Cat# B1793, USA, diluted in 0,1% DMSO) at DIV2 of the experiment after they have been exposed to three different NAM concentrations (0.1 mM, 0.2 mM and 0.4 mM) at DIV0. Bafilomycin A1 was added to the culture medium for 4 h, at a final concentration of 10 nM before cells were harvested for lysis. In all cases, control microglial cells were treated with the vehicle (70% EtOH).

After microglia detachment, the OPC population was separated from the underlying astrocytes by vigorous shaking (16 h at 240 rpm, 37°C). OPCs were then washed and seeded at an initial density of 35,000 cells per well in 48-well plates with 9 mm glass coverslips that were previously coated overnight with PDL (as above). Proliferation assays were performed as follows: cells were cultured in OPC culture medium [DMEM (GlutaMAX™, 4.5 g/L d-Glucose, -Pyruvate, Gibco, Cat# 61965-026), supplemented with 1% N2 (ThermoFisher Scientific, Cat#17502, USA), 60 μg/ml cysteine (Sigma-Aldrich, Cat# B4501, USA), 100 ng/ml biotin, 1% Penicillin/Streptomycin 10,000 units (Gibco, Cat# 15140-122), 0.1% BSA fatty acid-free (Sigma, A6003) containing 10 ng/ml PDGFα (Proteintech Cat No. HZ-1215) and 10 ng/ml hFGF (Proteintech Cat No. HZ-1103-GMP) to enhance the proliferation of OPCs. The day of cell plating, which is considered as DIV0, NAM diluted in 70% EtOH was added to the wells at three different concentrations (0.1, 0.2, and 0.4 mM) and cells were fixed at DIV3 for immunocytochemistry assays. Differentiation assays were performed as follows: the OPC culture medium was changed at DIV3 with new medium containing 40 ng/ml T3 (Sigma-Aldrich, Cat# T6397, USA), to allow the differentiation of OPCs toward mature oligodendrocytes. NAM was added to the wells at DIV3, as well, at a concentration of 0.4 mM and cells were fixed at DIV5 for immunocytochemistry assays. In all cases, control OPCs were treated with the vehicle (70% EtOH). After OPC detachment, the remaining astrocytes were washed 2 times with pre-heated PBS 1x. A total of 7 ml of trypsin (Gibco, Cat# 15090046) diluted in 1x PBS to a final concentration of 0.25% were added in the 75 cm^2^ culture flask for a maximum of 5 min, until astrocytes detached from the flask. Then, 8 ml of DMEM, supplemented with 10% FBS and 1% Penicillin/Streptomycin were inserted to the flask to inactivate trypsin and the suspension was collected and centrifuged at 1000 rpm for 5 min. After resuspension, astrocytes were maintained in DMEM, supplemented with 10% FBS and 1% Penicillin/Streptomycin and were seeded at an initial density of 200,000 cells per well in PDL-coated 24-well culture plates. The day of cell plating is considered as DIV0.

Nicotinamide (NAM) was added to the wells at three different concentrations (0.1 mM, 0.2 mM and 0.4 mM) at DIV0 of the experiment, whereas at DIV3 cells underwent starvation and were maintained in starvation medium (DMEM supplemented with 0.1% FBS and 1% Penicillin/Streptomycin) for 4 h. Afterward, starvation medium was removed and cells were treated with 100 ng/ml of LPS and different NAM concentrations (0.1 mM, 0.2 mM and 0.4 mM) in DMEM, supplemented with 10% FBS and 1% Penicillin/Streptomycin for 24 h, until they were collected for lysis. In another case, vehicle-treated and NAM (0.1 mM, 0.2 mM and 0.4 mM)-treated astrocytes were incubated with bafilomycin A1 at a final concentration of 10 nM at DIV2 for 4 h and they were harvested for lysis. For all treatments, control astrocytes were treated with the vehicle (70% EtOH).

#### 2.4.2. Immunocytochemistry

Cells were fixed using pre-heated 4% PFA (Sigma-Aldrich, Cat# 16005, USA) in 1x PBS for 10 min at 37°C. Washing with 1x PBS was followed by incubation of the cells in blocking solution of 1% BSA (PanReac AppliChem, Cat# A1391, USA), 0.1% TritionX-100 in 1x PBS for 30 min at RT. Afterward, cells were incubated with primary antibodies in blocking solution for 1.5 h at RT. A total of 10 min washing with 1x PBS was followed by labeling of the cells with the appropriate secondary fluorescent antibodies and DAPI (1:1,500, Invitrogen, Cat# MP01306, USA) in blocking solution for 45 min at RT. Finally, cells were washed in PBS for 10 min and coverslips were mounted with mounting medium containing Mowiol^®^ 4-88 Reagent (Millipore, Cat# 475904, Germany). The primary antibodies used were as follows: anti-IBA-1 (1:500, rabbit, Invitrogen, Cat# PA5-21274, USA), anti-PDGFRa (1:100, rat, Millipore Cat# CBL1366, Germany RRID:AB_11211998), anti-CC1 (1:100, mouse, Calbiochem Cat# OP80-100UG), anti-Ki67 (1:250, rabbit, ThermoFisher Cat# MA5-14520, USA),anti-PLP (1:1,000, rabbit, Abcam, Cat# ab28486). The following secondary antibodies were used: CF™ 488A goat anti-rabbit IgG (H + L) (1:800, Biotium, Cat# 20012, USA), CF488A goat anti-rat IgG (H + L) (1:800, Biotium, Cat# 20023, USA), Cy™ 3-conjugated goat anti-rabbit IgG (H + L) (1:800, Jackson ImmunoResearch, Cat# 111-165-003, UK), Alexa Fluor™ 555 goat anti-mouse IgG (H + L) (1:800, ThermoFisher, Cat# A-21422, USA). Images were obtained using confocal laser scanning microscopy (Leica microsystems, TCS SP8, Germany) and processed using Fiji image processing package (see text footnote 1).

#### 2.4.3. Enzyme-linked immunosorbent assay (ELISA)

Supernatants of vehicle-treated (70% EtOH) and nicotinamide (NAM)-treated primary microglia and astrocyte cultures were collected and used for cytokine measurements via sandwich ELISA. Cells underwent starvation for 4 h and after the removal of starvation medium they were treated with 100 ng/ml of LPS followed by NAM (0.4 mM) or vehicle treatment in DMEM, supplemented with 10% FBS and 1% Penicillin/Streptomycin for 24 h, when the supernatants were collected. Regarding microglia, IL-10 levels were measured using the ELISA MAX™ Deluxe Set Mouse IL-10 (Biolegend, Cat# 431414, USA), whereas in case of astrocytes, the levels of TNF-α were evaluated using the MAX™ Deluxe Set Mouse TNF-α (Biolegend, Cat# 430904, USA). For the evaluation of IL-10 in astrocytes, vehicle-treated (70% EtOH) and NAM-treated (0.1, 0.2, and 0.4 mM) supernatants were collected from primary cultures following incubation with Bafilomycin A1 for 4 h and the levels of IL-10 were measured using the ELISA MAX™ Deluxe Set Mouse IL-10 (Biolegend, Cat# 431414, USA). Three independent experiments were performed, following the manufacturer’s instructions and all samples were run in triplicates. Briefly, 100 μl of a mouse TNF-α or IL-10-specific capture antibody is coated on a 96-well plate, incubated overnight at 4°C and washed four times in at least 300 μl PBS–Tween-20 0.05%. A total of 200 μl of 1X assay diluent is used to block unspecific binding upon incubation for 1 h at room temperature (RT) with shaking. After four times-washing, 100 μl of standards and samples are added to the wells and incubated for 2 h at RT with shaking. Four time-washing of the plate is followed by the addition of 100 μl of biotinylated detection antibody for 1 h at RT with shaking. The plate is washed four times and 100 μl/well of avidin-horseradish peroxidase is subsequently added for 30 min at RT. After an extensive 5-time-washing of the wells, 100 μl of 3,3′,5,5′-Tetramethylbenzidine (TMB) substrate solution is added and the plate is incubated in the dark. The production of a blue color is proportional to the concentration of the examined cytokine that is present in the sample. Finally, 100 μl of stop solution is added to each well, changing the reaction color from blue to yellow. Absorbance is read at 450 nm and 570 nm within 15 min in a microplate reader, with the value at 570 nm being subtracted from the one at 450 nm.

#### 2.4.4. Western blot analysis

Cells cultured in 24-well plates were washed once in 1x PBS, collected in RIPA buffer [50 mM Tris-HCl pH 8.0, 150 mM NaCl, 1% Triton X-100, 0.5% sodium deoxycholate (DOC)] supplemented with protease inhibitor cocktail (Sigma-Aldrich, Cat# P8340, USA) and stored at −80°C. When used, they were thawed on ice, sonicated at 45 Hz and centrifuged (35 min, 11,000 rpm, 4°C). The total protein concentration in each sample was quantified using the Bradford kit (Bio-Rad Laboratories, Cat# 5000006). Protein samples were mixed with 4x laemmli buffer and dithiothreitol (DTT) 0.1 M (Sigma-Aldrich, Cat# D-9779, USA), heated at 95°C for 5 min and separated by sodium dodecyl sulfate polyacrylamide gel electrophoresis (SDS-PAGE) on an 8 or 12% polyacrylamide gel (gel thickness: 1.5 mm, 15 wells). Afterward, proteins were transferred to a nitrocellulose membrane (Sigma-Aldrich, Cat# 10600002, USA) for 1 h at 320 mA and stained with Ponceau S solution (Sigma-Aldrich, Cat# P7170, USA) to check transfer quality. The membrane was washed in PBST (PBS-Tween-20 0.1%) and after blocking for 1 h at RT in 3% BSA (PanReac AppliChem, Cat# A1391, USA), it was incubated in the primary antibodies, diluted in 3% blocking buffer, overnight, at 4°C. The following primary antibodies were used: anti-LC3B (1:1,000; rabbit, Sigma-Aldrich, Cat# L7543, USA), anti-iNOS (1:1,000; rabbit, Abcam, Cat# ab15323), anti-α-Tubulin, clone DM1A (1:5000; mouse, Sigma-Aldrich, Cat# T9026, USA). Three 15-min washes in PBST were followed by incubation of the membrane in secondary horseradish peroxidase-conjugated antibodies (1:5,000, Millipore, Cat# AP308P, AP132P, Germany) diluted in 3% blocking buffer for 1 h at RT. After three 15-min washes in PBST, blots were developed by chemiluminescence (Immobilon Classico Western HRP substrate, Millipore, Cat# WBLUC0500, Germany or Immobilon ECL Ultra Western HRP substrate, Millipore, Cat# WBULS0100 and WBULS0500, Germany). Quantification of band intensity was performed using the Image Lab Software (BioRad).

### 2.5. Statistical analysis

Statistical analysis was performed using GraphPad Prism 8 software (GraphPad Software, San Diego, CA, USA). In all experiments, quantification was performed using unpaired, parametric *t*-tests, since values follow a normal distribution, and data were expressed as mean ± SEM. *P*-values < 0.05 were considered statistically significant.

### 2.6. Quantification analysis

Densitometric analysis was used for the quantification of myelin basic protein (MBP), glial fibrillary acidic protein (GFAP), and IBA1 immunohistochemistry. For *ex vivo* quantification of myelinated axons Jacob plugin in Image J was used. Western blot analysis was performed with Image LAB.

## 3. Results

### 3.1. NAM is beneficial for myelination and remyelination *ex vivo*

**FIGURE 1:**
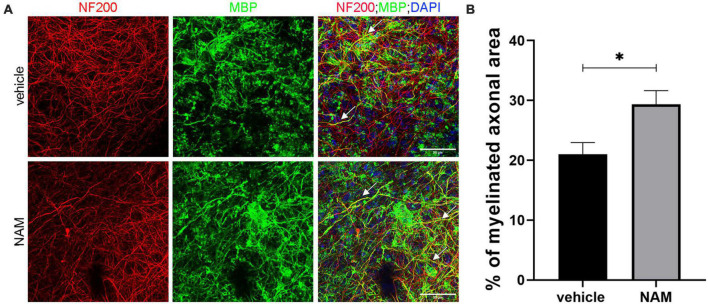
Nicotinamide (NAM) increases myelination of axons in *ex vivo* organotypic brain slices. **(A)** Representative confocal images of the cortical area of organotypic brain slices from postnatal day 4 mice, immunolabeled with an antibody against NF200 (red) to mark axons and an antibody against MBP (green) to mark myelin. DAPI was used for nuclear staining (blue). White arrows point at the myelinated axons (yellow). **(B)** Quantification of the percentage of NF200-positive area that co-localized with MBP, indicates the percentage of myelinated axons. Two groups were used: vehicle-treated (EtOH) for the control and NAM-treated (0.1 mM) for 6 days. For both groups *n* = 4. Data are shown as mean ± SEM. Student’s *t*-test was used to determine statistical significance. *p < 0.05. Scale bar: 50 μm.

Our first aim was to analyze the action of NAM *ex vivo*, so as to evaluate its ability to enhance myelin production under physiological conditions. Toward this aim, brain slices were treated with 0.1 mM NAM for 6 days, as described in the section “2. Materials and methods” and were then fixed and assayed for myelin basic protein (MBP, a myelin marker) and neurofilament 200 (NF200, an axonal marker) via immunohistochemistry. We observed increased co-localization of MBP on NF200+ axons in NAM-treated brain slices and an increase of myelinated axons upon NAM treatment (Figure 1). These results indicate that NAM enhances myelination in organotypic brain slices *ex vivo*.

**FIGURE 2:**
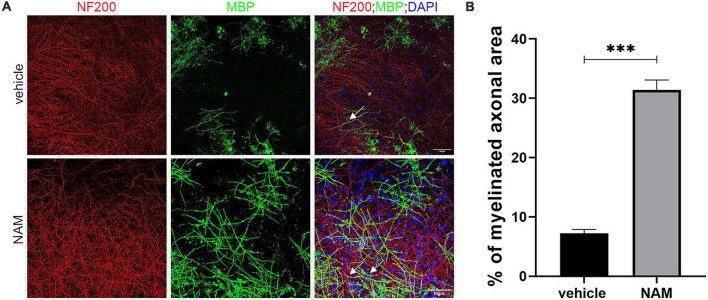
Nicotinamide (NAM) induces remyelination of axons in *ex vivo* organotypic brain slices after LPC demyelination protocol. **(A)** Representative confocal images of the cortical area of organotypic brain slices from postnatal day 4 mice, immunolabeled with an antibody against NF200 (red) to mark axons and an antibody against MBP (green) to mark myelin. DAPI was used for nuclear staining (blue). EtOH was used as control and compared with NAM treatment at 0.1 mM for 6 days. In both groups LPC demyelination protocol was applied prior to EtOH or NAM treatment. White arrows point at the myelinated axons (yellow). **(B)** Quantification of the percentage of NF200-positive area that co-localized with MBP, indicates the percentage of myelinated axons. Two groups were used: LPC and EtOH treatment for the control and LPC and NAM treatment at 0.1 mM for 6 days. For both groups *n* = 4. Data are shown as mean ± SEM. Student’s *t*-test was used to determine statistical significance. ***p ≤ 0.001. Scale bar: 50 μm.

We then evaluated the effect of NAM on remyelination upon LPC-induced demyelinating conditions *ex vivo*. LPC is an endogenous lysophospholipid which in high concentrations induces myelin damage through non-specific lipid disruption of the myelin membrane (Plemel et al., 2018). In our case, brain slices were incubated with LPC on day 14 of the culture protocol and were then returned to normal medium. Three days after LPC removal, when maximum demyelination had taken place (Eigel et al., 2019), slices were treated with 0.1 mM NAM for 6 days. Once the treatment was complete, brain slices were evaluated by immunohistochemistry against MBP and NF200. Our data revealed increased co-localization of markers MBP and NF-200, indicative of (partially) myelinated axons in NAM-treated brain slices, suggesting enhanced remyelination compared to control slices. In addition, the percentage of myelinated axons was enhanced upon NAM treatment (Figure 2). These results suggest that NAM enhances remyelination after LPC-induced demyelination in organotypic brain slices *ex vivo*.

### 3.2. NAM enhances myelin production following LPC-induced demyelination *in vivo*

As our data from the *ex vivo* studies suggested that NAM plays a beneficial role in remyelination, we decided to further investigate its effect under demyelinating conditions *in vivo*. To address this question, we used adult male C57BL/6 mice that were subjected to LPC-induced demyelination in the CC, which results in focal myelin loss with minimal axonal damage (Lu et al., 2018). This model is well-established and creates a focal demyelinating lesion with a specific temporal profile and well-defined borders (Blakemore and Franklin, 2008; Chu et al., 2019). The CC is the largest white matter structure of the brain that connects the two hemispheres via myelinated commissural fibers (Bénézit et al., 2015). Due to its high white matter concentration, it serves as an ideal target for focal demyelination studies in which the lesion area can be evaluated by myelin disruption and, as in our case, by increased cellular accumulation that is delineated by dotted lines in the figures.

**FIGURE 3:**
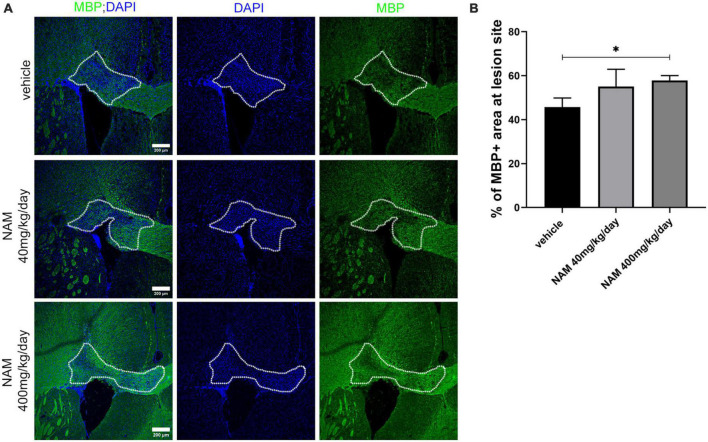
Nicotinamide (NAM) increases myelin production in the lesion site of the LPC-induced demyelination mouse model at 14 dpi. **(A)** Representative immunohistochemical confocal images of the corpus callosum labeled for MBP (green) and DAPI (blue). The area surrounded by dotted line denotes the lesion area. **(B)** Quantification of MBP density in control mice (LPC), in mice which received the lower dose of NAM after LPC (40 mg/kg/day) and in mice which received the higher dose of NAM after LPC (400 mg/kg/day) for 14 days. For all 3 groups *n* = 5. Data are shown as mean ± SEM. Student’s *t*-test was used to determine statistical significance. *p < 0.05. Scale bar: 200 μm.

Based on the existing literature, we decided to administer NAM at two different concentrations (40 and 400 mg/kg/day) and evaluate its effect on remyelination at 14 days post LPC-injection (dpi). This timepoint represents a stage in which the physiological process of remyelination has naturally taken over demyelination (El Waly et al., 2014). To assess if NAM treatment has an effect on myelin regeneration, we performed densitometric analysis of the demyelinated area upon MBP immunohistochemistry, as described in the section “2. Materials and methods.” Our results showed that NAM, when administered at the concentration of 40 mg/kg/day, had no effect on myelin density, in contrast to the higher dose that significantly increased MBP levels (Figure 3). We also investigated whether there was an effect of NAM administration at an earlier timepoint using only the higher dose of NAM, in order to implement the 3Rs principle. Specifically, we selected to analyze the effect of NAM at 7 dpi, a timepoint representing the peak of demyelination/initiation of remyelination (El Waly et al., 2014). Our results demonstrated that even at the early remyelination timepoint of 7 dpi, there is a significantly augmented myelin density (Supplementary Figure 1).

### 3.3. NAM affects microgliosis and astrogliosis upon LPC-induced demyelination *in vivo*, favoring myelin production

Lysolecithin (LPC) injection in the CC leads to activation and accumulation of both astrocytes and microglia in the lesion area, which, through the secretion of cytotoxic factors contribute to demyelination and mature OL loss (Plemel et al., 2018). Meanwhile, the accumulation of these two glial populations is followed by myelin debris clearance which eventually enables the remyelination process (Fan and Huo, 2021). Due to the significance of these two glial populations in myelin damage repair, we wondered how NAM will affect both microglia and astrocytes in this model.

**FIGURE 4:**
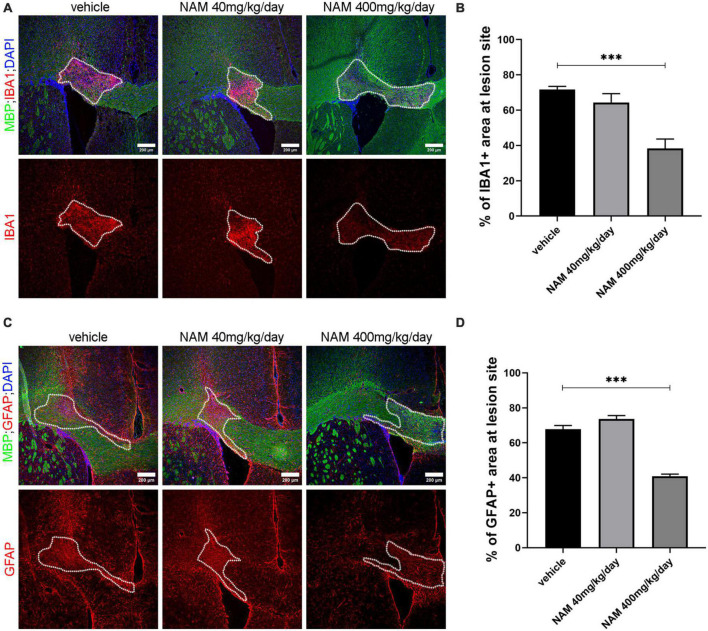
Nicotinamide (NAM) reduces both microgliosis and astrogliosis in the lesion area of LPC-induced demyelination at 14 dpi. **(A)** Representative immunohistochemical images of the corpus callosum labeled for MBP (green), IBA1 (red) and DAPI (blue). **(B)** Quantification of densitometric analysis for IBA1 in control mice (LPC), in mice which received the lower dose of NAM after LPC (40 mg/kg/day) and in mice which received the higher dose of NAM after LPC (400 mg/kg/day) for 14 days. **(C)** Representative confocal images of the corpus callosum labeled for MBP (green), GFAP (red) and DAPI (blue). **(D)** Quantification of densitometric analysis for GFAP in control mice (LPC), in mice which received the lower dose of NAM after LPC (40 mg/kg/day) and in mice which received the higher dose of NAM after LPC (400 mg/kg/day) for 14 days. The area surrounded by dotted lines denotes the lesion area. A total of 14 days of treatment after LPC stereotactic injection. Three groups of mice (vehicle with only LPC injection, NAM 40 mg/kg/day and NAM 400 mg/kg/day with LPC) were tested. For all groups *n* = 5. Data are shown as mean ± SEM. Student’s *t*-test was used to determine statistical significance. ***p ≤ 0.001. Scale bar: 200 μm.

First, we examined whether NAM affects microglial cell accumulation at the lesion site at 14 dpi. For this reason, we performed immunohistochemistry for the general microglial marker IBA1 on cryosections derived from all 3 different groups (vehicle, NAM 40 mg/kg/day and NAM 400 mg/kg/day). Densitometric analysis of IBA1 revealed a significant decrease of the microglial population at the lesion site (Figures 4A, B) in the group that received NAM at 400 mg/kg/day, which also had enhanced myelin immunoreactivity as shown by MBP staining. Reduced microgliosis shown by decreased IBA1 signal was also observed at 7 dpi at the lesion site upon NAM treatment (Supplementary Figure 2).

In parallel, in order to evaluate the effect of NAM on astrocytes we performed immunohistochemistry for the general astrocytic marker GFAP and quantified its levels by densitometric analysis. Astrocyte accumulation follows the same pattern with microglia. At 14 dpi there was no effect on astrocytic accumulation in animals treated with NAM at the concentration of 40 mg/kg/day. On the other hand, the higher NAM dose resulted in a significant decrease of astrocytes at the lesion site (Figures 4C, D). A significant decrease of astrocytic accumulation was also observed at 7 dpi (Supplementary Figure 3). Taken together, our data support the hypothesis that NAM accelerates the overall myelin production *in vivo*, by reducing both microgliosis and astrogliosis.

### 3.4. NAM treatment does not affect the oligodendrocytic lineage upon LPC-induced demyelination *in vivo*

Since OLs constitute the myelin-producing cells of the CNS and their proliferation and differentiation affects the remyelination process (Kuhn et al., 2019), we subsequently asked whether NAM has an effect on cells of the OL lineage following LPC-induced demyelination *in vivo*. To this end, we immunolabeled cryosections derived from vehicle-treated and NAM (400 mg/kg/day)-treated animals for oligodendrocyte markers that are expressed at different stages of the OL lineage in order to evaluate the effect of NAM on these populations. We selected to study the 7 dpi timepoint which was characterized by enhanced myelin density upon NAM treatment; thus, if there is an effect of NAM on the oligodendrocyte population we could detect it at this earlier timepoint. Specifically, the PDGFRa marker was used to assess the OPC population and the CC1 marker to detect mature OLs. Our analysis revealed no differences in the numbers of the different subpopulations between the two groups (vehicle and 400 mg/kg/day NAM-treated mice) after seven days of treatment (Supplementary Figure 4). We also analyzed the effect of NAM on oligodendrocyte linage cells *in vitro*, by performing primary cell cultures of OPCs derived from P2 mice. We observed no effect of NAM on proliferation or differentiation of OPCs *in vitro* (Supplementary Figures 6, 7).

### 3.5. NAM treatment reduces microglia activation *in vitro* in a dose-response manner

The *in vivo* system represents the most optimal model to evaluate the effect of a substance on an organism as, in this model, multiple cellular populations may interact and influence the effect of NAM on the remyelination process. To evaluate the direct effect of NAM on these specific populations which seem to be affected in the *in vivo* model, namely microglia and astrocytes, we performed *in vitro* primary glial cultures. We used a range of NAM concentrations from 0.1 to 0.4 mM, with the lower dose being the same as the dose in our *ex vivo* experiments.

**FIGURE 5:**
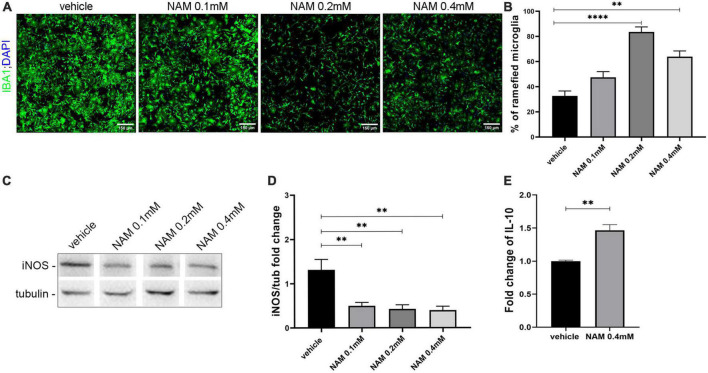
Nicotinamide (NAM) suppresses microglial activation *in vitro*. **(A)** Representative immunocytochemical confocal images of primary microglia cultures. Microglia labeled with IBA1 (green) marker and nuclear DAPI (blue). Four groups were used: vehicle, NAM 0.1 mM, NAM 0.2 mM, and NAM 0.4 mM. **(B)** Quantification of percentage of ramified microglia in the four different conditions. **(C)** Western blot analysis for iNOS in microglial lysates after activation with LPS. Four groups were used: vehicle, NAM 0.1 mM, NAM 0.2 mM, and NAM 0.4 mM. **(D)** Quantification of Western blot analysis for the four different conditions. **(E)** ELISA analysis for the secretion of anti-inflammatory cytokine IL-10. Two groups were used: vehicle and 0.4 mM NAM. For immunocytochemical experiments, *n* = 4. For biochemical experiments, *n* = 3. Data are shown as mean ± SEM. Student’s *t*-test was used to determine statistical significance. **p ≤ 0.01, ****p ≤ 0.0001. Scale bar: 150 μm.

First and foremost, we asked whether NAM could influence microglia activation *in vitro* (Figure 5). To test this hypothesis, we established primary microglia cultures derived from newborn P2 C57BL/6 mice, in which IBA1-positive cells were analyzed. Upon their activation, microglial cells undergo a series of morphological and functional changes. They are characterized by high plasticity and they actually switch from a ramified to an amoeboid morphology, a change that can easily be detected by immunocytochemistry for the IBA1 marker. LPS was used to activate the microglia and three different concentrations of NAM were used as described in “2. Materials and methods.” Our results indicated that NAM inhibits activation of microglia (Figures 5 A, B). Several studies have showed that activated microglia can be pro-inflammatory or anti-inflammatory (Kotter et al., 2001; Rawji and Yong, 2013; Nemes-Baran et al., 2020). In order to examine the effect of NAM on microglial activation state, we performed Western blot analysis for the inducible isoform of nitric oxide synthase, iNOS, a marker linked to a pro-inflammatory phenotype (Zeng et al., 2023) and ELISA assays for the anti-inflammatory marker IL-10. Cells were activated with LPS and three different concentrations of NAM were used. Our analysis revealed that iNOS levels decreased after NAM treatment, indicating that NAM effectively reduces the pro-inflammatory load of microglia, while it also induces their anti-inflammatory phenotype as indicated by the increased secretion of the anti-inflammatory cytokine IL-10 upon 0.4 mM of NAM treatment (Figures 5 C–E).

### 3.6. NAM treatment enhances the anti-inflammatory phenotype of astrocytes *in vitro*

Astrocytes can also be pro-inflammatory (A1) or anti-inflammatory (A2). A1 astrocytes can be identified by the pro-inflammatory cytokine TNF-α, while the A2 type of astrocytes can be identified by the anti-inflammatory cytokine IL10. Primary astrocyte cultures were performed and the supernatants were used for ELISA assays in order to evaluate the effect of NAM on A1 and A2 polarization.

**FIGURE 6:**
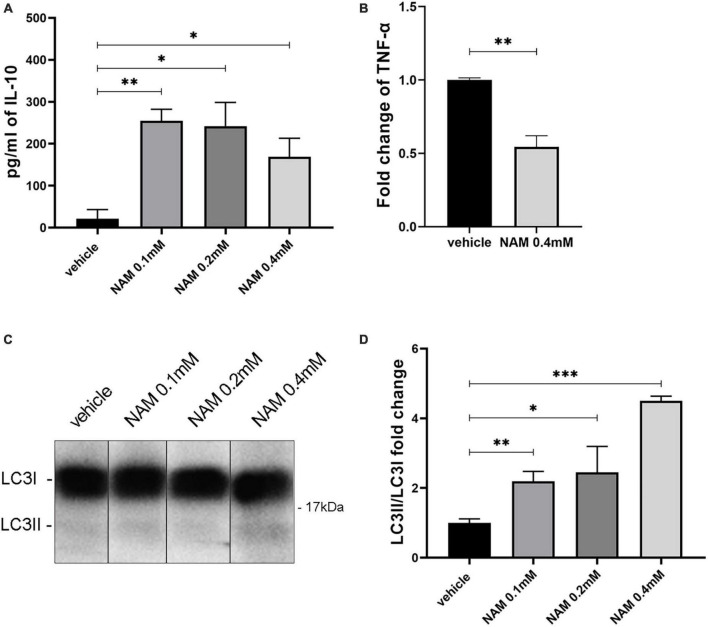
NAM induces both autophagy and the anti-inflammatory phenotype of astrocytes *in vitro*. **(A)** Quantification of IL-10 secretion from astrocytes after NAM treatment at concentrations of 0.1, 0.2, and 0.4 mM compared with the vehicle. **(B)** ELISA analysis for the secretion of the pro-inflammatory factor TNF-α in activated with LPS, primary astrocyte cultures. Two groups were used: vehicle and 0.4 mM NAM treated. **(C)** Western blot analysis for LC3 in lysates from primary cultures of astrocytes under four different conditions (vehicle, NAM 0.1 mM, NAM 0.2 mM, and NAM 0.4 mM). All groups were also treated for 4 h with 10 nM Bafilomycin A1 (BafA1) before cells were collected. **(D)** Quantification of LC3II/LC3I protein levels. For all procedures, in each group *n* = 3. Data are shown as mean ± SEM. Student’s *t*-test was used to determine statistical significance. *p < 0.05, **p ≤ 0.01, ***p ≤ 0.001.

Supernatants derived from four different groups containing three different concentrations of NAM and vehicle treatment, were tested for the expression of TNF-α and IL10. Our results showed an increase in the levels of IL-10 (Figure 6A). To detect TNF-α, the cells were activated with LPS, as described in the section “2. Materials and methods.” After treating the cells with 0.4 mM NAM for 24 h, the levels of the pro-inflammatory factor TNF-α were decreased compared to the control vehicle-treated cultures (Figure 6B).

### 3.7. NAM treatment induces autophagy in astrocytes *in vitro*

Since NAM constitutes an autophagy inducer, we sought to determine its role regarding this process in astrocytes and microglia. To be sure that we could detect its effect on autophagy, the V-ATPase inhibitor bafilomycin A1 was added to the culture medium as described at the section “2. Materials and methods.” Bafilomycin A1 disrupts the acidification of lysosomes and thus prevents the degradation of autophagic cargoes (Mauvezin and Neufeld, 2015). This effect results in the accumulation of proteins which participate in the autophagic machinery (such as LC3II) and thus it is considered an appropriate agent to better visualize the effect of a substance such as NAM, on autophagy. To this end, in our experiments we are using the ratio of LC3II/LC3I to evaluate the effect of NAM on autophagy (Kadowaki and Karim, 2009; Benischke et al., 2017). Four different conditions were tested (vehicle-treated, NAM 0.1 mM, NAM 0.2 mM and NAM 0.4 mM-treated). Western blot analysis with an antibody against the LC3 protein demonstrated higher levels of the LC3 II/LC3I ratio on the higher concentration of NAM (0.4 mM) compared to the other groups (Figures 6C, D), thus confirming that NAM induces autophagy directly in astrocytes. LC3-II is the autophagosome-membrane-associated lipidated form of microtubule-associated protein 1 light chain 3 (LC3/MAP1LC3), and is considered a reliable marker that associates with autophagosomes (Klionsky et al., 2016). Therefore, induction or suppression of autophagy is determined by monitoring the conversion of LC3-I to LC3-II in the presence of inhibitors of autophagosome-lysosome fusion, such as Bafilomycin A1 (Klionsky et al., 2016). Although NAM induces autophagy in astrocytes, there were no significant differences in the levels of LC3II/I between the different groups in microglia (Supplementary Figure 5).

## 4. Discussion

In the present study we have shown that NAM enhances myelination *ex vivo*, while it also induces remyelination. NAM treatment results in enhanced myelin production after LPC focal demyelination *in vivo*. The increased myelin immunoreactivity is accompanied by reduction in both astrogliosis and microgliosis. There is no effect of NAM on the oligodendrocyte lineage, as no differences are observed in OPC proliferation or differentiation or in the number of mature oligodendrocytes both *in vivo* and *in vitro*. However, we have noticed an effect on both microglia and astrocytes. More specifically, in primary cultures of microglia, NAM administration decreases the population of M1-activated microglia, while in primary cultures of astrocytes, an anti-inflammatory phenotype is induced as assayed by the production of IL-10 and the reduced expression TNF-α. Overall, we show that the increased myelin production that follows NAM treatment at both 7 and 14 dpi is accompanied by a decrease in both astrocyte and microglia accumulation at the lesion site. Our data indicate that NAM influences these populations directly, in favor of the remyelination process and by promoting a less inflammatory environment.

Nicotinamide (NAM) is a form of vitamin B3 and precursor of NAD^+^, a very important co-enzyme for redox reactions within the cell (Zhu et al., 2015), while it also serves as a CRM. Furthermore, NAD^+^ availability has been shown to affect the activity of several proteins associated with cell survival. One important protein family regulated by NAD^+^ concentration is the sirtuin deacetylase family of proteins (Kitada et al., 2016). Upon NAD^+^ supplementation, the sirtuin proteins are upregulated and trigger the transcription of key regulatory proteins for a multitude of metabolic or catabolic signaling pathways, gene expression, degeneration and cell death (Lee et al., 2008; Huang et al., 2015; Schultz and Sinclair, 2016). A major catabolic pathway which is directly or indirectly induced by the sirtuin pathway is autophagy. Sirtuin1 can induce the deacetylation of key regulatory proteins of the autophagic machinery such as Atg7, Atg5, and LC3, while it can also trigger the translocation of nuclear pools of LC3 to the cytoplasm (Lee et al., 2008; Huang et al., 2015).

There are several studies which also implicate NAM and its beneficial effect on remyelination. Wang and colleagues showed that administration of NAM enhances remyelination after stroke by promoting the maturation of OPCs (Wang et al., 2017). Another recent study using the EAE model of demyelination shows that NAD^+^ treatment results in fewer signs of myelin damage in the spinal cord and also shows that its effect is, at least in part, due to activation of autophagy (Wang et al., 2021). The most recent study, using the LPC model of demyelination, shows that NAM treatment in aged mice results in enhanced remyelination at the lesion site (Ma et al., 2022). The same group attributes this effect on OPC rejuvenation as NAD^+^ supplementation by β-NMN (the immediate precursor of NAD^+^) enhances both proliferation and differentiation of aged OPCs while only promoting differentiation of young OPCs *in vitro*. Additionally, in this study it was reported that this treatment influences myelin compaction/thickening as well as remyelination in aged mice. The mechanism proposed for the effects mentioned above is that NAD^+^ restores sirtuin 2 nuclear entry in the aged OPCs thereby delaying myelin aging. Therefore, it could be envisioned that since NAD^+^ supplementation enhances remyelination efficiency in the aged CNS, it could potentially be harnessed in translational studies for the treatment especially of progressive multiple sclerosis (MS).

Although the beneficial effects of NAM on myelin production have already been reported (Wang et al., 2017, 2021; Ma et al., 2022), the exact cellular populations, except oligodendrocytes, that are involved in the remyelination process and may be affected by NAM, are not studied yet. Thus, we sought to determine the putative effect of NAM on both microglia and astrocytes.

Here we determined the capacity of NAM to enhance myelin production with two different approaches, by using the *in vivo* LPC-induced model of focal demyelination and *ex vivo* organotypic brain slice cultures. The LPC-induced focal demyelination protocol is a well-established procedure where LPC injection leads to rapid disruption of lipid rich membrane structures (Plemel et al., 2018) such as myelin. Demyelination occurs at 3 dpi and the remyelination process starts at 10 dpi with a full recovery at 30 dpi under physiological conditions (Blakemore and Franklin, 2008). *Ex vivo* organotypic brain slice cultures are also a well-established protocol to monitor myelination during physiological or LPC-induced demyelinating conditions (Tan et al., 2018), while the *in vitro* approach allows the evaluation of the direct effect of NAM on specific cell populations, astrocytes and microglia in our case.

Both our *ex vivo* and *in vivo* experiments provide evidence that NAM treatment can increase the production of myelin under physiological conditions *ex vivo* and under LPC-induced demyelinating conditions *ex vivo* and *in vivo*. It is well established that the restoration of myelin requires the contribution and the interplay of all glial populations (Franklin and Ffrench-Constant, 2008; Kuhn et al., 2019). Specifically, the activation and accumulation of astrocytes, triggers the recruitment and activation of microglia to clear myelin debris. This step is critical for proper remyelination to take place and can be exerted by astrocytes as well (Skripuletz et al., 2013; Ponath et al., 2017). Then, since the environment is less inflammatory, the OPCs migrate, proliferate and differentiate at the lesion site in order to eventually myelinate axons. We show that after NAM treatment, there is a decrease in both microgliosis and astrocytosis at the lesion site *in vivo*. Our hypothesis is that this phenotype can limit the extent of the glial scar and lead to a less inflammatory microenvironment which favors new myelin production. It is worth mentioning that these two glial cell populations can have a pro-inflammatory or an anti-inflammatory phenotype. Therefore, in order to further elucidate their phenotype and clarify if their anti-inflammatory state outmatches their pro-inflammatory action, we performed *in vitro* primary cultures of both microglia and astrocytes.

Specifically for microglia, we first evaluated the action of NAM on their overall activation and afterward, the pro-inflammatory factor iNOS was examined by Western blot and the anti-inflammatory cytokine IL-10 was examined by ELISA assays, in primary cultures. The percentage of activated iNOS is a well-characterized marker of pro-inflammatory (M1) microglia (Luchena et al., 2022). After four days of treatment the levels of iNOS were significantly decreased after NAM treatment compared with the vehicle-treated cultures. Therefore, NAM affects the fate of microglia, reducing their pro-inflammatory phenotype. The increase of secreted IL-10 provides evidence that NAM treatment induces the anti-inflammatory phenotype of microglia (M2). Overall, the combination of both *in vitro* and *in vivo* experiments supports that NAM reduces the pro-inflammatory phenotype of microglia, an effect which could trigger the myelin debris clearance effectively and eventually enhance remyelination *in vivo*.

To elucidate whether NAM triggers the anti-inflammatory or the pro-inflammatory phenotype of astrocytes *in vitro*, we assessed the levels of IL-10 and TNF-α via ELISA assays. IL-10 is secreted by anti-inflammatory astrocytes and promotes the overall remyelination process, while TNF-α constitutes a well-established pro-inflammatory marker. The observed increase of IL-10 levels in NAM-treated cultures and reduction of TNF-α, in combination with the reduction of astrogliosis *in vivo*, suggests that NAM favors myelin production by inducing the anti-inflammatory phenotype of astrocytes and thus reducing inflammation at the lesion site.

Nicotinamide (NAM) is a CRM and thus an autophagy inducer (Yang and Zhang, 2020). Consequently, the beneficial effect of NAM on myelin production may be correlated with the induction of autophagy. To further investigate this possibility, Western blots against the LC3 protein were performed on both microglia and astrocyte primary cultures. Regarding microglia, our results provided evidence that there is no effect of NAM on the induction of autophagy. These results come in accordance with the existing literature as Pais and colleagues showed that Sirt2, a NAD^+^ dependent deacetylase, targets the deacetylation of transcription factor NF-κB, which plays a significant role in the regulation of inflammation. In the absence of Sirt2, NF-κB is hyperacetylated and thus, the transcription of pro-inflammatory factors is enhanced in microglia (Pais et al., 2013). Furthermore, increased levels of NAD^+^ result in Sirt2 upregulation (Wang et al., 2021). Taking everything into consideration, we could hypothesize that the suppression of the inflammatory phenotype of microglia is a result driven by enhanced Sirt2 expression in an autophagy-independent manner. On the other hand, our results indicated that NAM induces autophagy in astrocytes directly, in a dose response manner. Recently several studies showed that autophagy suppresses the inflammatory phenotype by degradation of inflammasomes and pro-inflammatory factors in astrocytes (Sacks et al., 2018). Thus, our data could suggest that the induction of autophagy through NAM treatment on astrocytes leads to the induction of their anti-inflammatory phenotype, a hypothesis that can be further investigated in future studies.

Overall, our data support that NAM triggers myelin production in the LPC focal demyelination model *in vivo* by providing a more favorable microenvironment for remyelination. Specifically, our data indicate that NAM may induce the anti-inflammatory phenotype of astrocytes and suppress the activation and pro-inflammatory phenotype of microglia.

## Data availability statement

The original contributions presented in this study are included in the article/Supplementary material, further inquiries can be directed to the corresponding author.

## Ethics statement

The animal study was reviewed and approved by the Animal Facility of the Institute of Molecular Biology and Biotechnology (IMBB)- Foundation for Research and Technology Hellas (FORTH) (license nos. EL91-BIObr-01 and EL91-BIOexp-02) complies with all regulations and standards outlined in the Presidential Decree 56/30.04.2013 (Greek Law). All procedures were performed in accordance with the EU directives and regulations (2010/63/EU and L 276/33/20.10.2010) that are equivalent to NIH standards established by the Animal Welfare Acts and the documents entitled “Principles for Use of Animals” and “Guide for the Care and Use of Laboratory Animals” from the Office of Laboratory Animal Welfare. Experimental animal protocols have been approved with license number 93164 (AΔA: 73XB7ΛK-ΦIΣ).

## Author contributions

SK designed and performed the *ex vivo*, *in vivo* experiments and analysis, designed the *in vitro* experiments, wrote the manuscript, and edited the figures. DeK performed the *in vitro* experiments and analysis, performed the Western blot and ELISA assays, and contributed to the writing of the manuscript. NK involved in the *in vitro* experiments, manuscript writing, and editing of the figures. AL performed some of the *in vivo* experiments and edited the manuscript. OK contributed to the design and performance of ELISA assays. MS designed experiments and was involved in data analysis. DoK designed and supervised the experiments and data analysis and wrote the manuscript. All authors contributed to the article and approved the submitted version.

## Footnotes

1
https://imagej.net/fiji
