# Supplementary material for: Nicotinamide enhances myelin production after demyelination through reduction of astrogliosis and microgliosis
Source: Front Cell Neurosci. 2023 Aug 17;17:1201317. doi: 10.3389/fncel.2023.1201317 (PMC10469866; doi:10.3389/fncel.2023.1201317)
Supplement: Supplementary file 1 [file Data_Sheet_1.docx]

Supplementary Material

**Nicotinamide enhances myelin production after demyelination through reduction of astrogliosis and microgliosis**

**Stefanos Ioannis Kaplanis, Despoina Kaffe, Niki Ktena, Andriani Lygeraki, Ourania Kolliniati Maria Savvaki, Domna Karagogeos^*^**

*** Correspondence:** Domna Karagogeos: karagoge@imbb.forth.gr

##
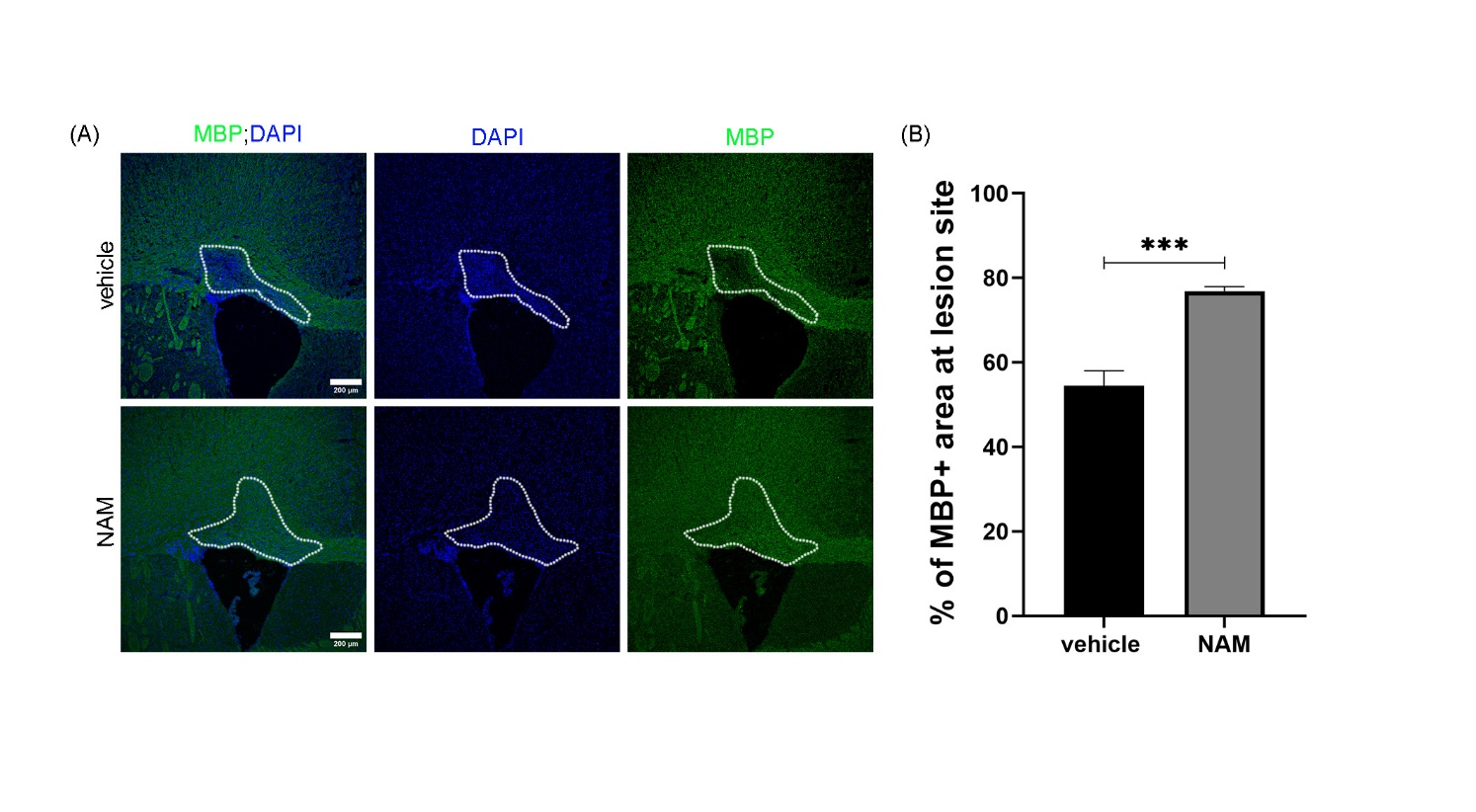


**Supplementary Figure 1.** NAM increases myelin production in the lesion area of the LPC-induced demyelination mouse model at 7dpi.

(A) Representative immunohistochemical confocal images of the corpus callosum labeled for MBP (green) and DAPI (blue). The area surrounded by dotted line denotes the lesion area. (B) Quantification of MBP density in control mice (LPC) and in mice which were treated with 400 mg/kg/day of NAM after LPC for 7 days. For both groups n=5. Data are shown as mean ± SEM. Student’s t-test was used to determine statistical significance., ***p ≤ 0.001. Scale bar: 200μm.


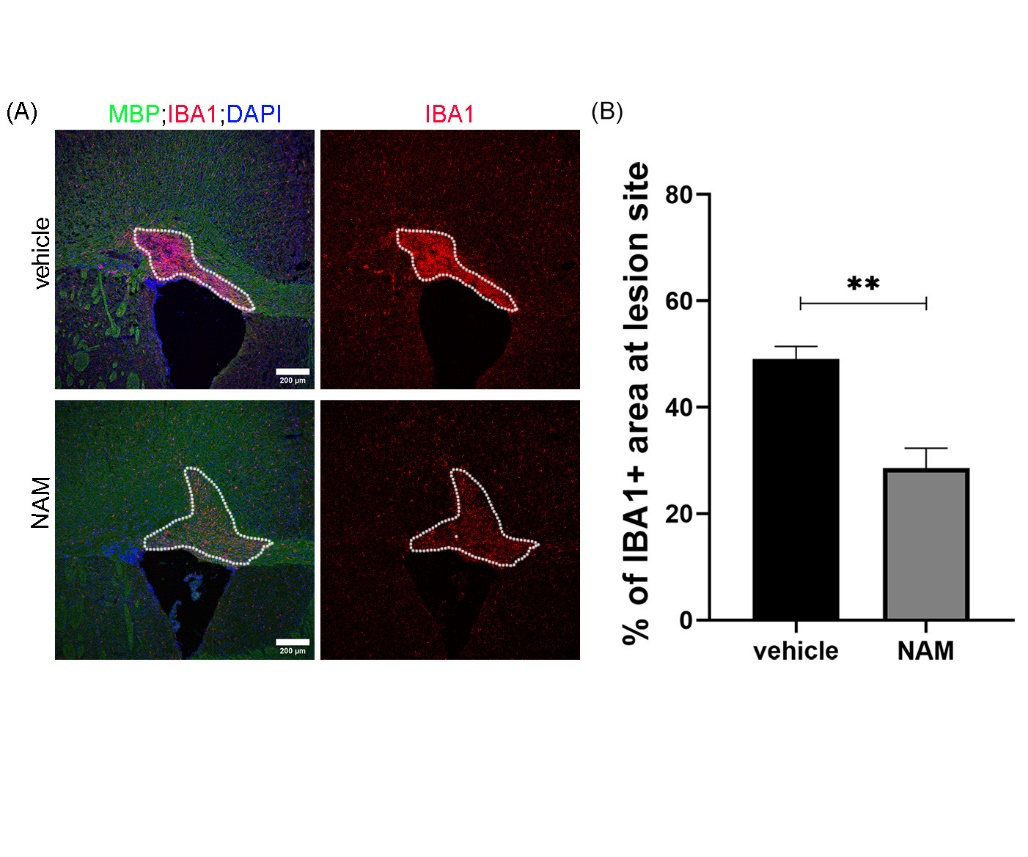


Supplementary Figure 2. NAM reduces microgliosis in the lesion area of LPC-induced demyelination mouse model at 7dpi.

(A) Representative immunohistochemical confocal images of the corpus callosum labeled for MBP (green), IBA1 (red) and DAPI (blue). The area surrounded by dotted line denotes the lesion area. 7 days of treatment after LPC stereotactic injection. Two groups of mice (vehicle with only LPC injection and NAM 400mg/kg/day with LPC and NAM treatment). (B)Quantification of densitometric analysis for IBA1 in control mice (vehicle) and in mice treated with 400mg/kg/day of NAM after LPC for 7 days. For both groups n=5. Data are shown as mean ± SEM. Student’s t-test was used to determine statistical significance. **p ≤ 0.01. Scale bar: 200μm.


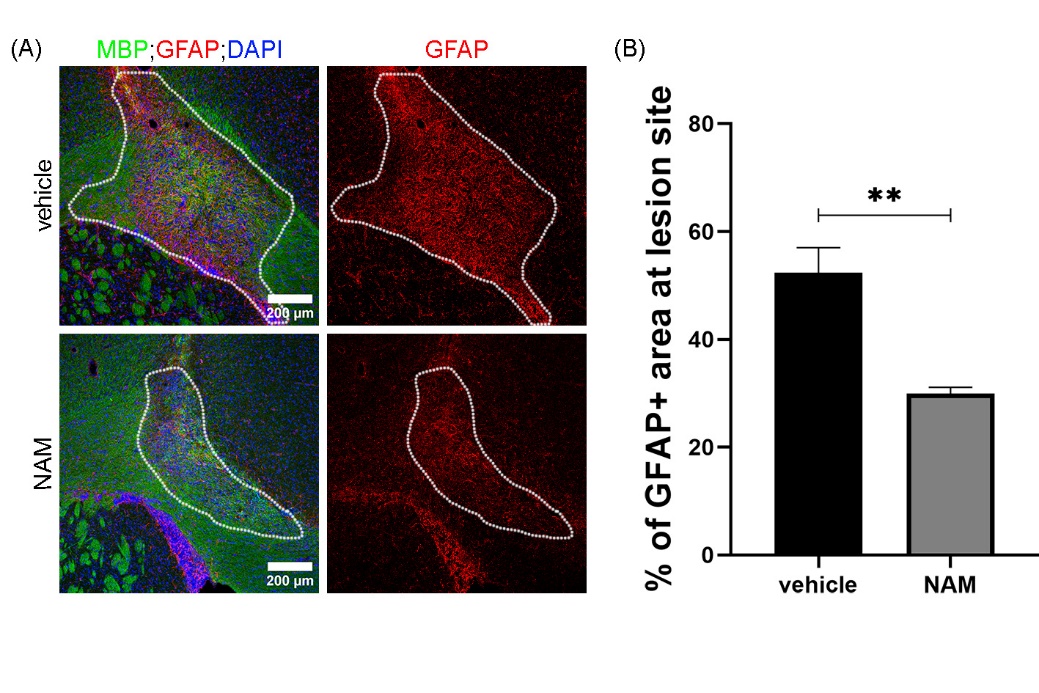


**Supplementary Figure 3.** NAM reduces astrogliosis in the lesion area of LPC-induced demyelination mouse model at 7dpi.

**(A)** Representative immunohistochemical confocal images of the corpus callosum labeled for MBP (green), GFAP (red) and DAPI (blue). The area surrounded by dotted line denotes the lesion area. 7 days of treatment after LPC stereotactic injection. Two groups of mice (vehicle only with LPC injection and NAM 400mg/kg/day with LPC. **(B)** Quantification of densitometric analysis for GFAP in control mice (vehicle) and in mice treated with 400mg/kg/day of NAM after LPC for seven days. For both groups n=5. Data are shown as mean ± SEM. Student’s t-test was used to determine statistical significance, **p ≤ 0.01. Scale bar: 200μm.


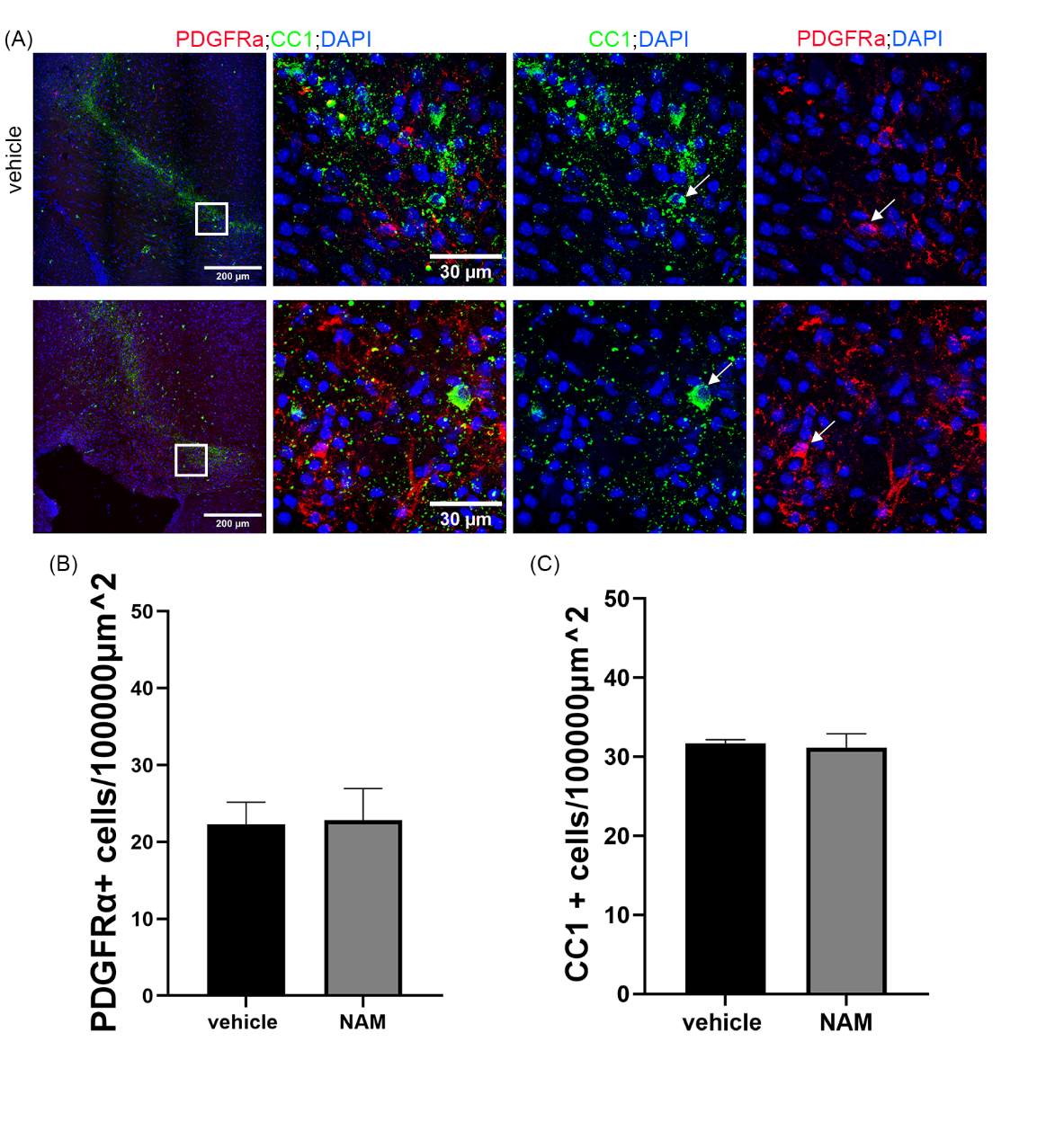


Supplementary Figure 4. NAM treatment does not affect the population of oligodendrocyte lineage at lesion site.

(A) Representative immunohistochemical confocal images of the corpus callosum labeled for CC1(green), PDGFRa (red) and DAPI (blue). Rectangular boxes indicate areas magnified to the right. (B, C) Quantification of PDGFRa and CC1-positive cells respectively per area in control mice (LPC) and in mice treated with 400mgr/kg/day NAM after LPC for 7 days. For both groups n=5. Data are shown as mean ± SEM. Student’s t-test was used to determine statistical significance. Scale bar: 200μm and 30μm.


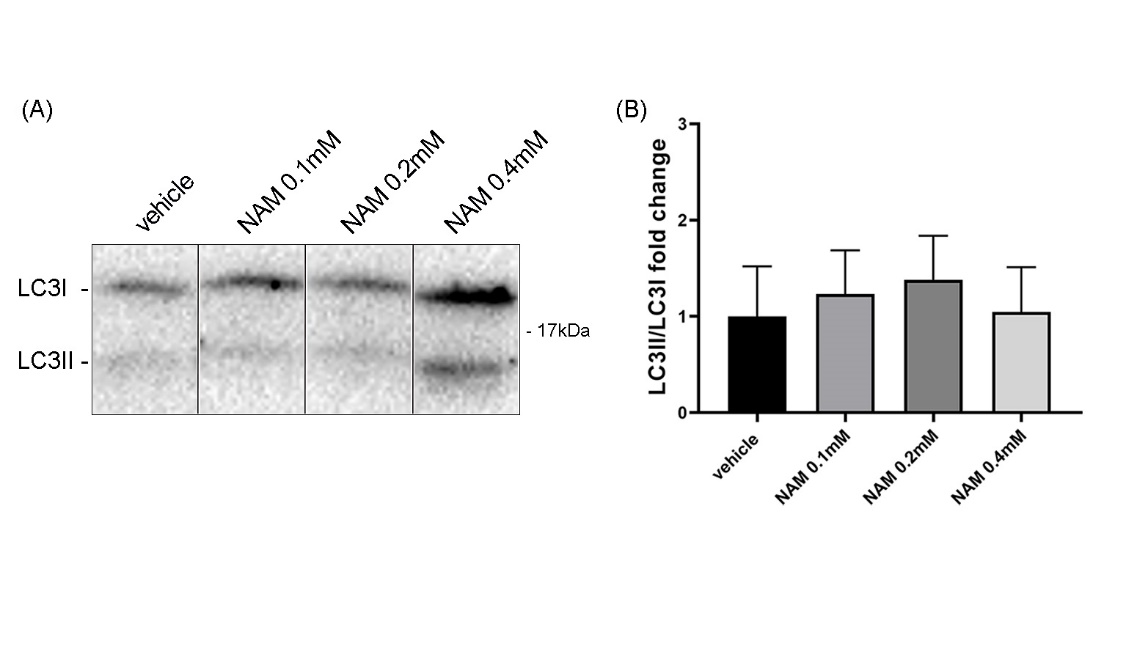


Supplementary Figure 5. NAM does not influence autophagy in microglia *in vitro*.

(A) Western blot analysis for LC3 in lysates from primary cultures of microglia under four different conditions (vehicle, NAM 0.1mM, NAM 0.2mM & NAM 0.4mM). All groups were also treated for 4 h with 10nM BafilomycinA1 (BafA1) before cells were collected. (B) Quantification of LC3II/LC3I protein levels. For all groups n=3. Data are shown as mean ± SEM. Student’s t-test was used to determine statistical significance.


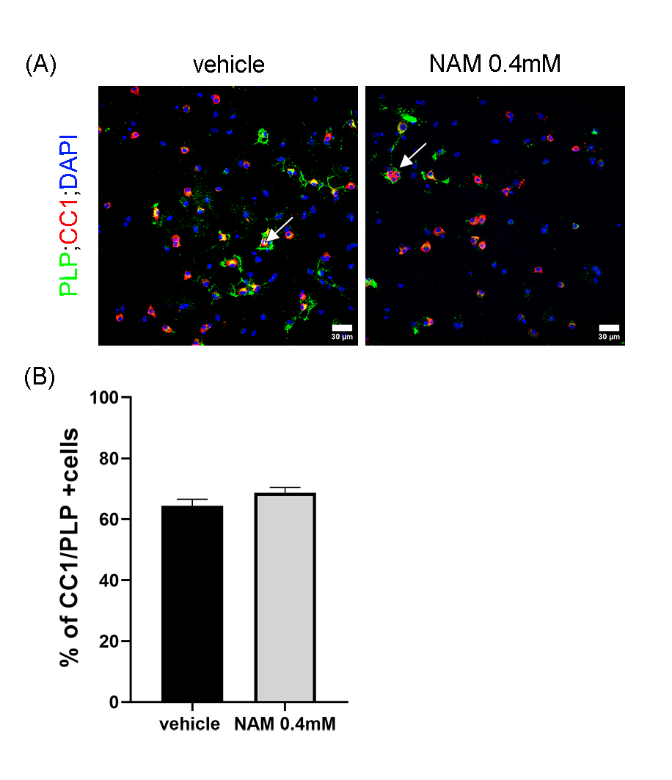


Supplementary Figure 6. NAM does not affect OPC differentiation *in vitro*

(A) Representative confocal images of OPC cultures after 2 days in differentiation medium labeled for CC1(red), PLP (green) and DAPI (blue). White arrows point to the double-stained cells. (B)Quantification of the percentage of PLP cells that are also positive for CC1 immunoreactivity. Two groups were used: vehicle and 0.4mM NAM treated. For both groups n=3. Data are shown as mean ± SEM. Student’s t-test was used to determine statistical significance. Scale bar: 20μm.


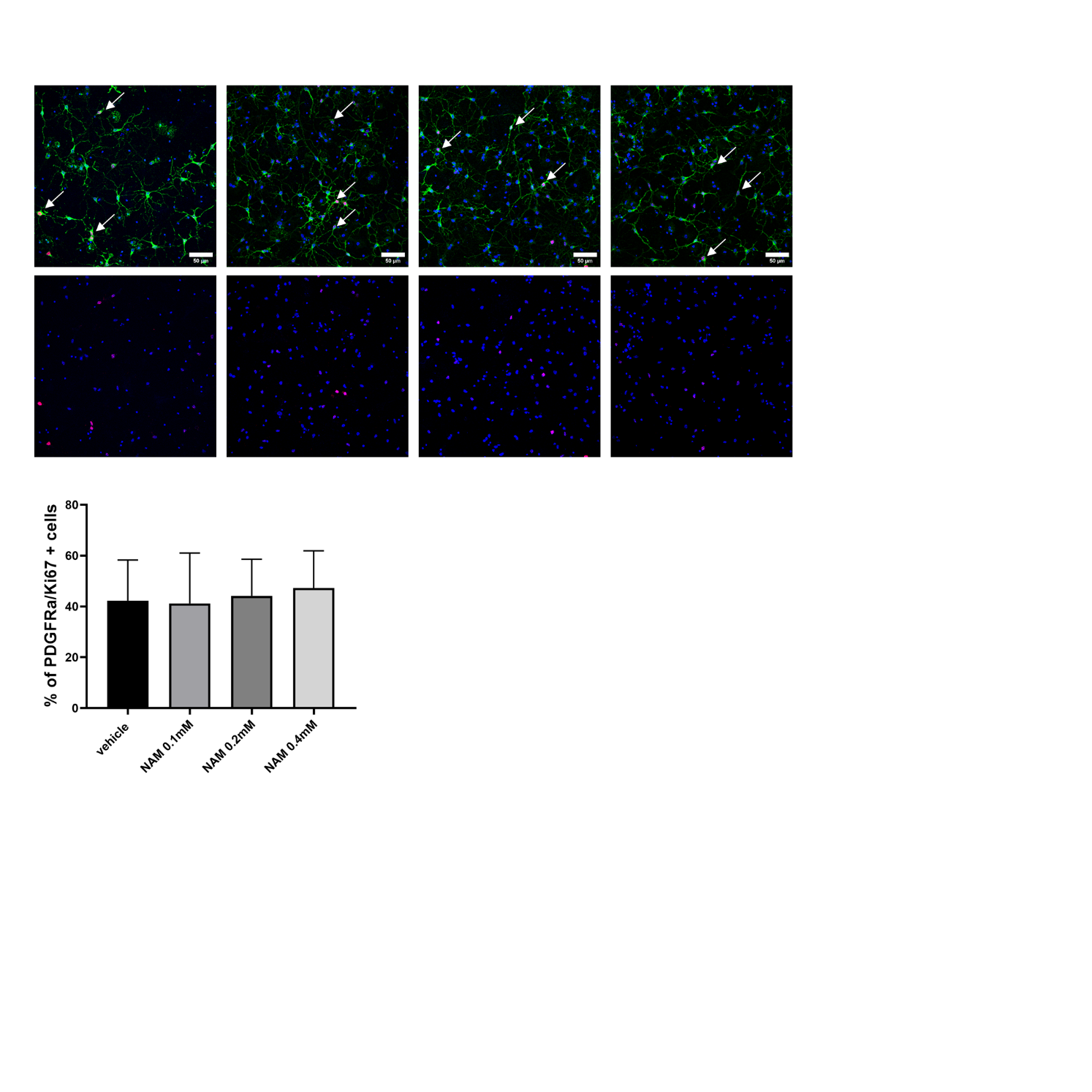


Supplementary Figure 7: NAM does not affect the proliferation of OPCs *in vitro.*

A) Representative confocal images of primary OPC cultures. OPCs labeled with PDGFRa (green), Ki67 (red) and nuclear DAPI (blue). Four groups were used: vehicle, NAM 0.1mM, NAM 0.2mM & NAM 0.4mM. (B) Quantification of percentage of double-stained cells for PDGFRa and Ki67, which represent the proliferating OPCs (white arrows point to double-stained cells), in the four different conditions. For all groups n=3. Data are shown as mean ± SEM. Student’s t-test was used to determine statistical significance. Scale bar: 50μm.
